# Feedback Can Be Less Stressful: Medical Trainee Perceptions of Using the Prepare to ADAPT (Ask-Discuss-Ask-Plan Together) Framework

**DOI:** 10.7759/cureus.3718

**Published:** 2018-12-11

**Authors:** Tyra Fainstad, Adelaide A McClintock, Monica J Van der Ridder, Susan S Johnston, Kristen K Patton

**Affiliations:** 1 Internal Medicine, University of Washington, Seattle, USA; 2 Medical Education and Simulation, Michigan State University, Grand Rapids, USA; 3 Medical Education and Simulation, University of Washington, Seattle, USA; 4 Cardiology, University of Washington, Seattle, USA

**Keywords:** feedback, feedback, coaching, qualitative, educational alliance

## Abstract

Introduction

Meaningful feedback is essential for effective medical education, yet the feedback process has been consistently problematic for both learners and faculty. Emerging research on feedback highlights the importance of the learner, relationships, and culture for feedback to improve performance. We used the theory of self-regulated learning to develop the Prepare to Ask-Discuss-Ask-Plan Together (Prepare to ADAPT) framework to improve the feedback processes and investigated learner perceptions of this innovative feedback framework.

Methods

Qualitative thematic analysis of structured interviews of nine trainees participating in training on the Prepare to ADAPT feedback framework.

Results

The framework appeared primarily to potentially decrease learner anxiety and stress around the feedback process by providing a simple, structured discourse pattern. We identified five contributing themes: (1) increased efficiency of the feedback process; (2) formation of coaching/teamwork relationships; (3) facilitation of reflection and goal identification; (4) increased frequency of the feedback; (5) increased usefulness of the feedback.

Discussion

The Prepare to ADAPT framework may help decrease stress and anxiety of the feedback by clarifying the process, applying a structure, and developing coaching relationships. The framework was found to be easy to use and increased the number of effective feedback conversations in this exploratory study.

## Introduction

Meaningful feedback is fundamental for effective medical education and ultimately, for improving patient care [1-3]. Unfortunately, the complexity of the feedback process remains consistently problematic for learners and faculty [4-5]. Learners are not satisfied with either the quantity or quality of feedback they receive, despite substantial attention to improving feedback provision via faculty development [6-9]. In 2014, our academic center’s Graduate Medical Education (GME) Office identified a need to improve feedback in our clinical learning environment. We sought to respond to an identified lack of frequent, useful feedback in clinical training by creating a learner-centered model to guide effective feedback conversations. Informed by the literature, we developed a five-step framework called Prepare to Ask-Discuss-Ask-Plan Together (Prepare to ADAPT) [10] designed to harness effective feedback practices and embed them into our training system.

In developing the framework, we relied on the emerging data that have identified barriers to the feedback process. Over the past decade, seminal research on feedback has clarified the importance of an effective feedback process as one that is motivated by improving performance and focused on the gap between a trainee’s performance and the desired standard [11]. Noting that framing of feedback as a one-way delivery of content has not led to an improved feedback culture, the “educational alliance” model reorients feedback conversations as a negotiation within the setting of a supporting educational relationship [12]. This emphasis on the social and cultural factors and placing the learner in a central role in the process is aligned with the standard educational strategies to improve the engagement of the adult learner.

Adding to the complexity of creating an effective feedback process, learner performance has been noted to be influenced by many, and sometimes conflicting factors, including personal relationships, prior knowledge, emotions, cultural norms, and previous experiences [13]. A successful example of the feedback process improvement that guided us by constructively employing these concepts is the R2C2 (Relationship, Reaction, Content, Coaching) feedback model, which is based on a humanistic approach guided by behavior change theory and the concept of informed self-assessment [14].

The culture of medicine has fostered infrequent direct observation of the clinical skills, despite requirements by accrediting bodies, and the current understanding of the improved credibility of feedback afforded by observation [12]. The time demands on faculty and trainees do not encourage direct observation and a focus on learner autonomy and performance evaluation over formative feedback further discourage this effective technique [15].

A landmark article from organizational psychology adds to the understanding of barriers around feedback and focuses on the learner role in seeking and receiving feedback. Ashford et al. describe two types of learner goal orientation: (1) performance oriented (i.e. “look smart”) and (2) learning oriented (i.e. “improve”) [16]. The achievement-based, high stakes, competitive medical learning environment may induce a performance goal orientation which can lead to avoidance of the essential feedback [17]. Fostering a “growth” or learning-based mindset increases receptivity to feedback and feedback seeking behavior and supports the development of mastery [18-19].

Other pivotal work on feedback has converged on the positive consequence of self-reflection and developing a plan for improvement based on a feedback conversation. Goal setting, in particular, is an effective strategy to improve the effects of feedback on performance [20]. A coaching conversation on an observed performance, directed towards a learning goal, with reflection on the performance and a standard by the learner harnesses these valuable insights into how to improve feedback [21].

Building upon the existing discourse-based model of “Ask-Discuss-Ask”[22], we grounded the Prepare to ADAPT model in a conceptual framework of self-regulated learning with a goal-setting phase, a performance phase, and a reflection and planning phase [23]. Our goals included the following: 1) promoting a learner-centered educational environment and enhancing psychological safety to foster relationships between learner and teacher [24]; 2) developing learner goal identification, self-reflection and feedback-seeking skills; 3) increasing the number of feedback encounters with an efficient framework; and 4) highlighting lifelong learning skills and the development of an improvement plan. An online learning module was created to educate learners on how to use the framework [25].

We conducted an exploratory qualitative evaluation study of residents and fellows to increase our understanding of “how and why” the Prepare to ADAPT Framework affects the learners’ feedback experiences and their ability to identify areas of their performance to improve upon. Our central research question is: what are learners’ perceptions of engaging with the Prepare to ADAPT Framework, and was the framework useful for improving the feedback process?

## Materials and methods

Study setting and participant selection

**Figure 1:**
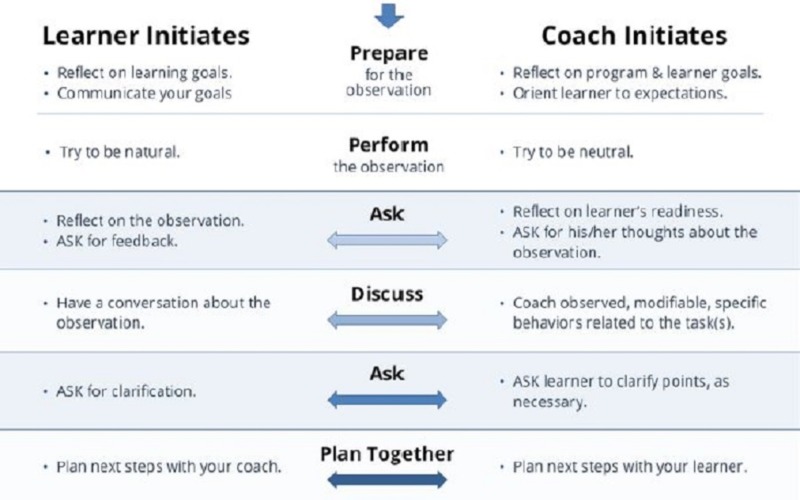
Prepare to ADAPT feedback framework

Our institution is a large academic teaching system. Our research team was composed of three clinician educators (KP, TF, AHM) and two educators (SSJ, JMMvdR) who developed the feedback framework: Prepare to ADAPT (Figure 1). Between February and December 2016, a pilot group of residents and fellows (*n *= 36) were invited to complete the Prepare to ADAPT online learning module [25]. As this was an exploratory study, our sample was a convenience sample: some of the participants were invited by the members of the research team or were invited as members of our institution’s Trainee Curriculum Advisory Committee. Twelve trainees completed the module and were invited to be interviewed. Our institution’s Human Subjects Division Institutional Review Board reviewed the study and judged it to be educational program improvement and not research. 

Data collection

Investigators jointly designed the semi-structured interview guide. Interview questions were based on literature that stimulated the initial development and objectives of the Prepare to ADAPT Framework. This method allowed us to explore how the framework was used and valued (or not) in practice [26]. Questions were structured around eliciting trainee perspectives about the usefulness of the Prepare to ADAPT Framework and ease of use of the online module. Informed consent was obtained from each participant. An experienced interviewer (SSJ) who did not previously know the participants conducted in-person, individual semi-structured interviews, ranging from 20 to 50 minutes. Interviews were held in locations convenient to the trainees and field notes were taken. In total, nine trainees completed interviews. All interviews were audio-recorded, transcribed verbatim (SSJ), and de-identified. Two team members conducted a second review of transcriptions (KKP, TF) to control for transcription errors.

Analysis

The transcripts were uploaded to Dedoose online (www.dedoose.com), a software program designed to facilitate coding and qualitative analysis. The data were analyzed using qualitative evaluation of themes through constant comparison. The available literature on feedback and communication theories [27] guided us in the process of analyzing the data and deriving themes via open, axial, and selective coding [26]. Open coding was conducted by four team members (TF, KKP, AHM, and SSJ) to define a preliminary framework for the raw data using phrases or sentences as the units of analysis. When JMMvdR joined the team, the data were re-coded by all team members. Raw data were chunked to develop a coding tree (Table 1). We progressed into the phase of axial coding by relating codes to each other and discovering various dimensions. In the selective coding phase, we focused on the description of our interpretation of the data. Discrepancies were resolved through a process of deliberation until consensus was achieved. We were aware of each researcher’s characteristics throughout the process as a consideration of reflexivity. The Consolidated Criteria for Reporting Qualitative Studies (COREQ) was used to report our findings. The outcomes of our analysis were sent back to our participants, referred to as “learners” below, for a member check to provide evidence for the validity of our outcomes [28].

**Table 1:** Example of a coding tree in the early phase of open coding

Codes	Number of quotes
Sender, recipient, and relationship	
Negatives of a hierarchy	3
Roles of different people. This can be both positive and negative	20
Context	
Current system	1
Positive attributes to the current system	21
Culture change	41
Communication	
Coaching conversation	29
Barriers to effective feedback	72
Challenges of giving	15
Barriers to asking	10
Framework	
Problems with the framework	7
Benefits of framework	64
Content	
Challenges to ‘Prepare’ step of framework	17
Challenges to ‘Plan Together’ step	9
Benefits of ‘Prepare’ step	27
Benefits of ‘Plan Together’ step	16
Benefits of ‘asking’ steps	6
Benefits of ‘Ask-Discuss-Ask’ steps	3
Miscellaneous	
Remnants (miscellaneous and don’t know)	18

## Results

**Figure 2:**
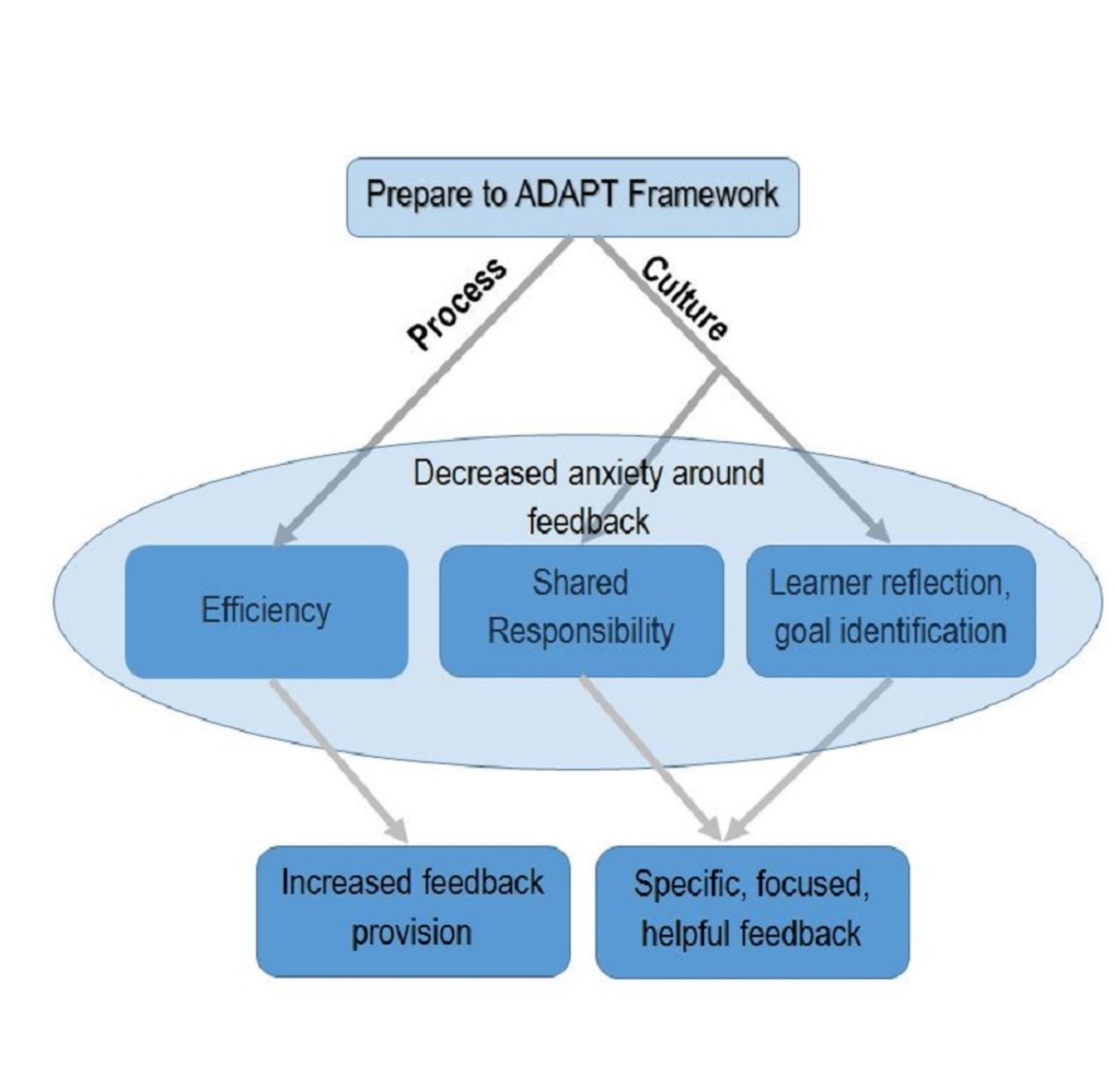
Conceptual model of Prepare to ADAPT Conceptual model of proposed utility of the Prepare to ADAPT feedback framework according to the learners’ perception

Nine interviews were conducted with trainees of various specialties (Table 2). The main theme emerging from our data is that the Prepare to ADAPT framework reduced anxiety around the process of obtaining feedback in the clinical learning environment (Figure 2). Five additional supporting themes arose: efficiency of the framework, a paradigm shift towards feedback as coaching, fostering of learner reflection and goal-setting, increased feedback provision, and a more useful, focused feedback.

**Table 2:** Participant demographic characteristics

Demographic characteristics	No
Training Level	
Resident	4
Fellow	5
Specialty	
Internal Medicine	2
Physical Medicine and Rehab	1
Cardiology	2
Emergency Medicine	3
Obstetrics and Gynecology	1
Gender	
Female	5
Male	4
Mean age (years)	31

Reduction of anxiety

Nearly all participants reported that using the framework reduced the anxiety often associated with receiving feedback. Within this context, many learners reported that the framework provided a structure that helped clarify expectations, was easy to remember, and reduced the risk of “the ask.” Related to this, several participants mentioned that they thought the framework reduced the stress of the attending and suggested that the stress reduction might increase the frequency of feedback-seeking behavior.

 “I like the structure; I like the approach [of having a mnemonic], and it is easy to remember.” (Learner 2)

“Honestly, just having this structure is probably the biggest plus [...] it takes away the anxiety for both people.” (Learner 7)

“[Using the framework] feels like a less stressful way to give feedback—kind of wrapping it up nicely.” (Learner 4)

“I think [the framework] reduces the stress level of the attending which makes [the learner’s] job easier, because I am more likely to ask for [feedback] if I know it is not perceived as an ordeal… the attendings don’t have to think ‘Uh oh, I need to backtrack and think through a month’s worth of clinical encounters to give this learner feedback.’ So I think it made [my attendings] a lot more comfortable.” (Learner 9)

Efficiency

Participants reported that the Prepare to ADAPT Framework was relatively easy to incorporate into daily practice and made the feedback process easier. Specifically, learners remarked that the model was efficient and was not perceived as a ‘burden’.

“The framework was fast also - it took just a couple of minutes. It was [...] as fast as feedback can be. It was not burdensome.” (Learner 4)

“Removes all the barriers—not time intensive, not overwhelming in terms of scope. [The framework adds] simplicity in terms of incorporating into your day-to-day clinic and having it be useful without it being too cumbersome to remember.” (Learner 6)

 “How much extra time? 10 minutes total to do [the framework] in clinic. Not much time at all; [it was] ideal to have my attending right there; really easy to facilitate it.” (Learner 1)

Paradigm shift: coaching and teamwork relationship

Learners reported that the model helped them work together with their attending during the process of receiving feedback. This theme of the shared responsibility for the feedback process frequently co-occurred with the theme of anxiety reduction. 

“I kind of liked thinking of attendings in a coach way, as it doesn’t feel hierarchical. As coaches, they are there to help you. Working on things together. I liked the coach idea—it is more encouraging…What this model offers is “We are all in this together [...] how can we all improve. Gets everyone involved in the process. Let’s all come up with things to help us improve. Brings residents and attendings together [...] it doesn’t feel hierarchical...” (Learner 4)

Learner 1 echoed this sentiment of teamwork and coaching as a “lower stakes” relationship: “I like the coach terminology. Easier to ask for a coach, like in sports, rather than a mentor. It is less formal. I like that.”

Learner 7 explained that the model was a kind of “leveler” that changed the relationship with his attending: “Having told [my attending], ‘Hey [can we try this framework]’ - it opened up a different sort of relationship. This tool was an ice-breaker and it made [feedback] go better.” 

“Using this framework makes it a more useful feedback [conversation] when there isn’t much continuity [...] you have that type of feedback opportunity where you don’t necessarily require long relationships.” (Learner 9)

Learner reflection and goal identification

Almost all learners mentioned that the framework stimulated them to reflect on their own knowledge gaps and learning goals. Specifically, the “Prepare” and the “Plan Together” steps encouraged learners to recognize and define their own needs.

Learner 9 touched on how the model might promote meta-cognitive skills in the “prepare” step: “That self-analysis or introspection of ‘What do I need to work on today?’ [In the Prepare step] is a good learning process.” 

Similarly, participants reported that the Plan Together step helped clarify and promote commitment to the next steps for the learner.

“I particularly like the Planning Together part. Not only what you say [...] but now also what you can think of to do better next time. Planning it together makes it a two-way street: ‘here’s what I’ll do next time, with my attending helping and participating’.” (Learner 4)

“Because [of the Plan Together step] I had to think about it, and I had to kind of characterize [my learning need] and recognize it for what it was” (Learner 8)

Increased provision of feedback

Learners reported that using the framework helped foster and better recognize feedback conversations and allowed for feedback even when a learner did not have continuity with a given attending.

“The outcome from the framework is the individual getting more feedback and improving performance and patient care.” (Learner 1)

Learner 8 reported that the model leads to more feedback seeking and recognition when feedback was given: “[Right now, learners] feel they don’t know how to ask for feedback or they don’t recognize it when it is given to them. Having a framework is smart and helpful and making it part of normal interactions with people is a cool way to help with that.” 

Learners 8 and 9 also reported that the framework made feedback seem more “do-able” even when a resident-attending pair doesn’t have continuity, a common situation in medical training.

 “We hear it all the time, all the way through training: ‘Ask for feedback, make sure you get feedback, midterm, end-of- quarter.’ But I don’t think anyone has ever taught me how to do that [like this framework does]. That’s kind of a big deal.” (Learner 8)

“Using this framework makes it a more useful feedback [conversation] when there isn’t much continuity [...] you have that type of feedback opportunity where you don’t necessarily require long relationships.” (Learner 9)

Useful and specific feedback

Learners reported that the model helped them obtain more focused, specific, and useful feedback. This theme frequently co-occurred with statements regarding the use of the “prepare” and “plan together” steps.

“Most of the time [the Plan Together step] is me thinking about what I did and can do better specifically.” (Learner 4) 

“[The attending] is cued and primed [on the] expectation afterwards to give feedback, I think they are a lot more focused on that because they know what you are focused on.” (Learner 9)

In summary, using the Prepare to ADAPT Framework appears to reduce anxiety and stress created by the feedback process in the clinical learning environment. This concept is supported by the emerging themes both instrumental (efficiency, increased frequency, and increased utility), and interactive or constructivist (relationship, coaching, reflection and goal identification).

## Discussion

A growing body of literature confirms the problematic historical framing of feedback as a one-way provision of information from attending to the trainee. Current, more nuanced, understandings of the role of feedback in behavior change in adult learners reflect the complexity of human relationships, environment, and multisource time demands. We incorporated several concepts inherent to an effective feedback to develop a structured framework for feedback conversations. Beginning with a shift to a learner-centered model of feedback aligned with principles of adult learning, we developed the framework to be useful from either a feedback giver or seeker standpoint. We highlighted the importance of “preparing”, which allows for identification (guided or not, as needed) of a learning goal, and reflection on and then in action. Additionally, the initial conversation (“ask”) is structured to foment the development of a positive coaching relationship – an educational alliance. The “performance” aspect reinforces the importance of direct observation, which allows for more credible and useful feedback. The “ask-discuss-ask” conversation is designed to be driven by the learner, yet allows for the reinforcement or correction by the feedback-giver. Development of the action plan in the final step closes the cycle and promotes lifelong learning habits for identifying the next goal and creating a plan for improvement.

Our exploratory analysis of learner perceptions of using Prepare to ADAPT showed the framework may improve the feedback process by addressing several common barriers to feedback. The most striking theme that developed from the analysis was a reduction in anxiety and stress related to the feedback process from the use of a simple conversational framework. Secondary themes of efficiency, frequency, and usefulness related to the technical aspects of the feedback process; the influence of social interactions, culture, and constructivism were evident in the themes of a shift to a coaching relationship with a shared responsibility for feedback, reflection on and in action, and learner goal identification with the development of improvement plans.

Using the framework appears to create a shared mental model and a common structure for the feedback conversation, setting clear expectations, and improving transparency. This is important since the initial “ask” in seeking feedback is often reported in the literature as the most stressful part of the feedback process for learners [16-17]. The Prepare to ADAPT Framework ties the initial request for feedback to a learning goal, provides a purpose and language for the request, and thereby relieves the attending of the burden of retrospectively searching for a potential area for improvement. Feedback then becomes a shared responsibility for both the learner and the attending and encourages a supportive educational alliance [12]. Importantly, the framework centralizes the essential role of direct observation in the provision of feedback, often lost in the current climate of high volume, efficient patient care [7].

By shifting to a conversational mode where the feedback-giver is directed to focus on a learning goal identified by, or agreed to by the learner, the framework may reduce stress commonly associated with feedback as evaluation, and instead promotes the concept of feedback as a normal part of the learning and process, similar to how a coach is expected to guide performance improvement for a musician or athlete [12,15]. The conversational nature of the framework and the “plan together” step appeared to potentially change the dynamic from “teacher and student” to “team.” By encouraging learners to take an active role in feedback initiation and direction, Prepare to ADAPT may help transform the feedback process from a one-way, top-down, hierarchical “telling” into an interactive conversation between learner and attending with defined and agreed upon goals [2]. We hypothesize that this paradigm shift may lower the stakes for learners and be one of the mechanisms through which feedback becomes both less anxiety provoking and normalized [13].

Several learners identified simply having a structure as a particularly meaningful mechanism of reducing the anxiety around feedback. Specifically, the structure helped guide the conversation and limited it to the learning goals from the “Prepare” step. By keeping the conversation focused, it also allowed for efficiency and a perception of it being quick to employ. Given that time is one of the most commonly cited barriers to the feedback process [27], an unambiguous expectation of a short and goal-focused feedback conversation may have reduced concerns regarding the time required to engage in the feedback process. This aspect may help users to implement focused feedback conversations quickly and regularly within the busy clinical teaching environment.

Because of its “quick and easy” nature, our interviewees also commented on the potential of the model to increase the amount of feedback conversations that occur in the learning environment. They reported that asking for and receiving regular feedback was easier to accomplish with Prepare to ADAPT in their toolkit. In an era of duty hour limits, educators must strive to incorporate efficient and effective feedback strategies to meet competency goals. Prepare to ADAPT provides a tool to address this.

Learners remarked that the framework also improved the usefulness of the feedback. Of particular interest, learners clearly identified that using Prepare to ADAPT fostered metacognitive skills and the associated behavior change by providing regular practice identifying learning goals and “next steps” in their own learning. The framework may cultivate learner reflection and self-regulated learning. Goal identification is an important aspect of this framework that sets it apart from more traditional models in which teachers provide feedback based on what they believe the learner should know [20]. We propose that using this framework regularly will help learners refine this important skill, encourage them to seek critical feedback, and, ultimately, drive their own learning. Ultimately, we hope that using this framework may shift learner goals from performance-based orientations (i.e. look smart) toward learning-based (i.e. improve) [16-17].

Limitations

Our small sample size is small and therefore risks the lack of generalizability of the results. However, it is our sense that the identified themes are promising and worthy of future study. By describing our process of data collection and the interview guidelines (SM 1), by including the COREQ principles as they apply to this study (SM 3), and by conducting a member check on ‘resonance of results’ with our interviewees (SM 2), we believe this study meets the criteria of dependability. Responses from active participants that the outcomes resonated with them indicate the emerging themes can be seen as credible [28]. Another limitation is that we were only able to explore learner’s perceptions of the framework, and the study does not provide insight into whether the resulting feedback leads to actual performance improvement. However, the study is a starting point, because we do know that perceptions often determine behavior [29]. In addition, although we were able to interview trainees in a variety of different specialties, the sample lacks junior trainees; this is important since we postulate that earlier learners may have a harder time identifying essential learning goals.

## Conclusions

We sought to address the common barriers to effective feedback through the development of a usable learner-centered, conversational framework grounded by self-regulated learning theory. Our results suggest the use of the Prepare to ADAPT framework may enhance the feedback process by reducing anxiety and stress caused by feedback conversations. Additional themes of efficiency, utility, and frequency supported the ease of use, while social-cultural constructs of relationships, reflection, and goal identification emerged as fundamental supports of the feedback process. Using this tool may serve to facilitate an effective feedback in the clinical training environment, promote a positive feedback culture by creating coaching relationships, and advance the development of life-long learning skills critical to the profession.
